# *N*-(2-Hydroxy­ethyl)-2-[3-(*p*-tol­yl)triazen-1-yl]benzamide

**DOI:** 10.1107/S1600536809011908

**Published:** 2009-04-08

**Authors:** Fernando Rocha-Alonzo, Gerardo Aguirre, Miguel Parra-Hake

**Affiliations:** a Centro de Graduados e Investigación del Instituto Tecnológico de Tijuana, Apdo. Postal 1166, 22500, Tijuana, BC, Mexico

## Abstract

In the solid state, the structure of the title compound, C_16_H_18_N_4_O_2_, is stabilized by inter­molecular N—H⋯O and O—H⋯O hydrogen bonds. These hydrogen bonds arrange the mol­ecules into a double-layer supra­molecular structure. The mol­ecular conformation is is consolidated by an intra­molecular N—H⋯N hydrogen bond. The dihedral angle between the aromatic rings is 8.01 (10)°

## Related literature

For the synthesis of new ligands to stabilize dinuclear complexes and control their reactivity, see: Das *et al.* (2008 ▶); Estevan *et al.* (2006 ▶); Jie *et al.* (2007 ▶); Müller & Vogt (2007 ▶); Schilling *et al.* (2008 ▶). For the synthesis of 1,3-bis­(ar­yl)triazenes as precursors for triazenido ligands bearing Lewis basic *ortho* substituents such as ester, meth­oxy and methyl­mercapto groups, see: Nuricumbo-Escobar *et al.*(2007 ▶); Ríos-Moreno *et al.* (2003 ▶); Rodríguez *et al.* (1999 ▶); Tejel *et al.* (2004 ▶). The starting material 2-[4,5-dihydro-1,3-oxazol-2-yl]aniline was synthesized by a modification of the literature method of Gómez *et al.* (2005 ▶). For bond-length data, see: Allen *et al.* (1987 ▶); Orpen *et al.* (1989 ▶).
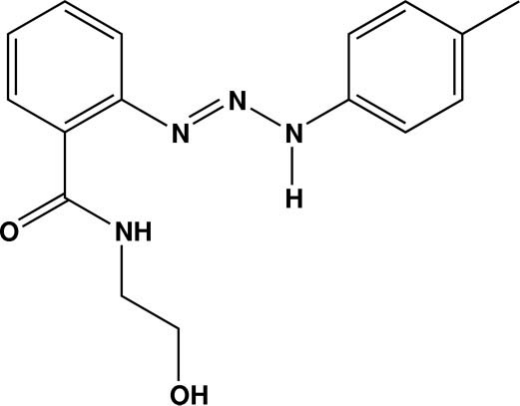


## Experimental

### 

#### Crystal data

C_16_H_18_N_4_O_2_
*M* *_r_* = 298.34
Monoclinic, 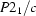

*a* = 16.846 (2) Å
*b* = 12.2053 (17) Å
*c* = 7.4302 (11) Å
β = 93.212 (13)°
*V* = 1525.3 (4) Å^3^
*Z* = 4
Mo *K*α radiation
μ = 0.09 mm^−1^
*T* = 298 K
0.40 × 0.22 × 0.14 mm

#### Data collection

Bruker P4 diffractometer
Absorption correction: none
4153 measured reflections
3067 independent reflections
1778 reflections with *I* > 2σ(*I*)
*R*_int_ = 0.044
3 standard reflections every 97 reflections intensity decay: 2.8%

#### Refinement

*R*[*F* ^2^ > 2σ(*F* ^2^)] = 0.055
*wR*(*F* ^2^) = 0.188
*S* = 1.04
3067 reflections
201 parameters
H-atom parameters constrained
Δρ_max_ = 0.57 e Å^−3^
Δρ_min_ = −0.22 e Å^−3^

### 

Data collection: *XSCANS* (Siemens, 1996 ▶); cell refinement: *XSCANS*; data reduction: *XSCANS*; program(s) used to solve structure: *SHELXS97* (Sheldrick, 2008 ▶); program(s) used to refine structure: *SHELXL97* (Sheldrick, 2008 ▶); molecular graphics: *Mercury* (Macrae *et al.*, 2006 ▶); software used to prepare material for publication: *SHELXL97*.

## Supplementary Material

Crystal structure: contains datablocks I, global. DOI: 10.1107/S1600536809011908/kp2210sup1.cif

Structure factors: contains datablocks I. DOI: 10.1107/S1600536809011908/kp2210Isup2.hkl

Additional supplementary materials:  crystallographic information; 3D view; checkCIF report

## Figures and Tables

**Table 1:** Hydrogen-bond geometry (Å, °)

*D*—H⋯*A*	*D*—H	H⋯*A*	*D*⋯*A*	*D*—H⋯*A*
N4—H4*A*⋯N1	0.86	2.05	2.696 (3)	132
O2—H2*B*⋯O1^i^	0.82	1.92	2.729 (2)	169
N3—H3*A*⋯O2^ii^	0.86	2.00	2.851 (2)	170

Symmetry codes: (i) 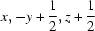; (ii) 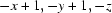.

**Fig. 1.**
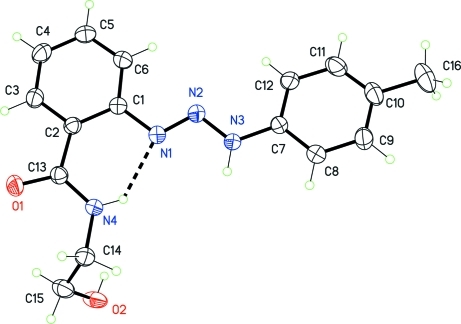
The title compound (I) with displacement ellipsoids drawn at the 30% probability level. Intramolecular H-bond is indicated by dashed lines.

**Fig. 2.**
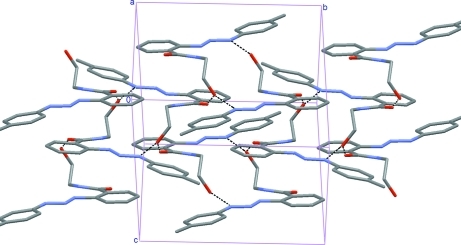
Packing of I showing the H-bonds. The molecules are forming a two dimensional network in the (100) plane. H-bonds are indicated by dashed lines.

**Fig. 3.**
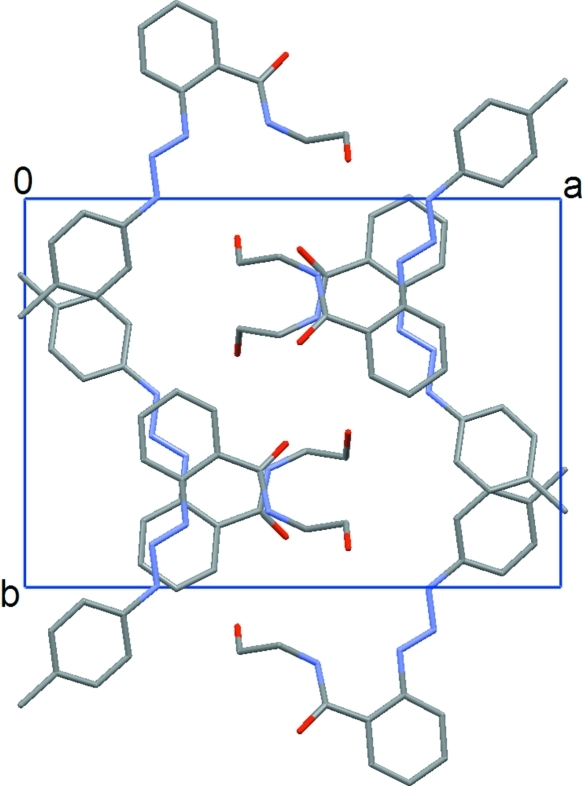
Packing of I showing the bilayer. The molecules are forming a zig-zag array along the [001] direction.

## supplementary crystallographic information

### Comment

The synthesis of alternative ligands to stabilize dinuclear complexes and control their reactivity is an area of great importance in coordination and organometallic chemistry (for recent literature see: Das *et al.*, 2008; Estevan *et al.*, 2006; Jie *et al.*, 2007; Müller & Vogt, 2007; Schilling *et al.*, 2008). In this context, we have focused our attention to the synthesis of 1,3-bis(aryl)triazenes as precursors for triazenido ligands bearing Lewis basic *ortho* substituents such as ester, methoxy and methylmercapto groups (Nuricumbo-Escobar *et al.*, 2007; Ríos-Moreno *et al.*, 2003; Rodríguez *et al.*, 1999; Tejel *et al.*, 2004); it has been found that the nature of the substituent produces a dramatic impact on their coordination chemistry and reactivity. As part of our ongoing research, we have synthesized the title compound (I, Fig. 1) using the diazonium salt *N*-coupling methodology.

The molecular structure of (I) shows the characteristic *trans* stereochemistry about N═N  of the diazoamino group of free triazenes. The N1═N2 bond [1.264 (3) Å] is longer than the typical value for N═N bond (1.222 Å), whereas the N2—N3 bond [1.320 (3) Å] is shorter than typical value for a Nsp^3^—Nsp^2^ single bond (1.420 Å) (Allen *et al.*, 1987). In addition, the C7—N3 bond [1.395 (3) Å] is shorter than the characteristic C_aryl_—NH single bonds for secondary aromatic amines (1.419 Å) (Orpen, *et al.*, 1989). An intramolecular N1—H···N4 hydrogen bond is observed (Fig. 1 and Table 1).

In the crystal structure, adjacent units are arranged into a two-dimensional network along the (100) plane *via* intermolecular N— H···O and O—H···O hydrogen bond interactions (Fig. 2 and Table 1). These layers are linked together *via *intermolecular N—H···O and O—H···O hydrogen bonds forming a zig-zag bilayered array along the [001] direction (Fig. 3).

### Experimental

The synthesis of the title compound included reagents and solvents of reagent grade, which were used without further purification. As a starting material we synthesized 2-[4,5-dihydro-1,3-oxazol-2-yl]aniline by a modification of the Gómez and coworkers methodology (Gómez *et al.*, 2005). 2-[4,5-Dihydro-1,3-oxazol-2-yl]aniline (1.00 g, 6.17 mmol) was dissolved in aqueous HCl 2 *M* (9.25 ml, 18.50 mmol) and cooled to 268 K. A sodium nitrite solution (0.51 g, 7.40 mmol) in water (6 ml) was slowly added with continuous stirring. A solution of *p*-toluidine (0.66 g, 6.17 mmol) in methanol (10 ml) was added slowly to the reaction mixture, and stirred for 30 m at 268 K. The resulting mixture was neutralised with a saturated aqueous solution of NaHCO_3_. A crude yellow-orange was separated by filtration and washed with small portions of water. The product was purified by flash chromatography on neutral alumina (hexane/ethyl acetate, 1:9), and recrystallized from an ethyl acetate/hexane mixture (9 : 1). Orange bar-shaped crystals of (I), suitable for X-ray analysis, were obtained by slow evaporation of the solvent mixture. Yield 47% (0.87 g, 2.90 mmol), based on 2-[4,5-dihydro-1,3-oxazol-2-yl]aniline; m.p., 111–113 °C. IR (KBr pellet, cm^-1^), 3278, 3233, 1625, 1538, 1269.^1^H NMR [(CD_3_)_2_CO, 200 MHz] δ 12.89 (*s*), 8.10 (*s*), 7.93–7.02 (m, 8H), 4.10 (*s*), 3.74 (dd *J *= 5.4, 11.0 Hz, 2H), 3.54 (dd, *J *= 5.4, 11.0 Hz, 2H), 2.35 (s, 3H).^13^C NMR [(C D_3_)_2_CO, 50 MHz] δ 135.4, 133.0, 130.1, 128.4, 121.7,114.9, 61.2, 43.2, 21.0. *Anal.* Calcd. for C_16_H_18_N_4_O_2_: C, 64.41; H, 6.08;N, 18.78%. Found C, 64.11; H, 6.44; N, 18.93%. HRESIMS Calcd. for [*M*+H]^+^299.1503. Found 299.1519.

### Refinement

Refinement for H atoms was carried out using a riding model, with distances constrained to: 0.93 Å for aromatic CH, 0.98 Å for methine CH. Isotropic U parameters were fixed to *U*_iso_(H)=1.2*U*_eq_(carrier atom) for aromatic CH.

### Crystal data

**Table:**

C_16_H_18_N_4_O_2_	*F*(000) = 632
*M**_r_* = 298.34	*D*_x_ = 1.299 Mg m^−^^3^
Monoclinic, *P*2_1_/*c*	Mo *K*α radiation, λ = 0.71073 Å
Hall symbol: -P 2ybc	Cell parameters from 76 reflections
*a* = 16.846 (2) Å	θ = 4.7–12.0°
*b* = 12.2053 (17) Å	µ = 0.09 mm^−^^1^
*c* = 7.4302 (11) Å	*T* = 298 K
β = 93.212 (13)°	Neele, yellow
*V* = 1525.3 (4) Å^3^	0.40 × 0.22 × 0.14 mm
*Z* = 4	

### Data collection

**Table:**

Bruker P4 diffractometer	*R*_int_ = 0.044
Radiation source: fine-focus sealed tube	θ_max_ = 26.3°, θ_min_ = 2.1°
graphite	*h* = −20→20
2θ/ω scans	*k* = −15→1
4153 measured reflections	*l* = −9→1
3067 independent reflections	3 standard reflections every 97 reflections
1778 reflections with *I* > 2σ(*I*)	intensity decay: 2.8%

### Refinement

**Table:**

Refinement on *F*^2^	Secondary atom site location: difference Fourier map
Least-squares matrix: full	Hydrogen site location: inferred from neighbouring sites
*R*[*F*^2^ > 2σ(*F*^2^)] = 0.055	H-atom parameters constrained
*wR*(*F*^2^) = 0.188	*w* = 1/[σ^2^(*F*_o_^2^) + (0.1035*P*)^2^ + 0.0651*P*] where *P* = (*F*_o_^2^ + 2*F*_c_^2^)/3
*S* = 1.04	(Δ/σ)_max_ < 0.001
3067 reflections	Δρ_max_ = 0.57 e Å^−^^3^
201 parameters	Δρ_min_ = −0.22 e Å^−^^3^
0 restraints	Extinction correction: *SHELXL97* (Sheldrick, 2008), Fc^*^=kFc[1+0.001xFc^2^λ^3^/sin(2θ)]^-1/4^
Primary atom site location: structure-invariant direct methods	Extinction coefficient: 0.008 (3)

### Fractional atomic coordinates and isotropic or equivalent isotropic displacement parameters (Å^2^)

**Table:**

	*x*	*y*	*z*	*U*_iso_*/*U*_eq_	
N1	0.69940 (11)	0.33793 (15)	−0.0017 (3)	0.0484 (5)	
N2	0.76320 (11)	0.39013 (16)	0.0332 (3)	0.0481 (5)	
N4	0.54570 (10)	0.30472 (15)	−0.1084 (3)	0.0456 (5)	
H4A	0.5790	0.3480	−0.0525	0.055*	
O1	0.51316 (10)	0.13062 (14)	−0.1750 (2)	0.0574 (5)	
O2	0.39653 (10)	0.39859 (16)	0.0593 (3)	0.0651 (6)	
H2B	0.4341	0.3834	0.1299	0.098*	
N3	0.75322 (11)	0.49670 (16)	0.0124 (3)	0.0521 (6)	
H3A	0.7066	0.5219	−0.0175	0.063*	
C1	0.70741 (13)	0.22274 (19)	0.0146 (3)	0.0434 (6)	
C2	0.64042 (13)	0.15801 (18)	−0.0313 (3)	0.0424 (6)	
C3	0.64717 (15)	0.0445 (2)	−0.0087 (4)	0.0550 (7)	
H3B	0.6029	0.0006	−0.0346	0.066*	
C4	0.71719 (17)	−0.0036 (2)	0.0504 (4)	0.0646 (8)	
H4B	0.7200	−0.0792	0.0651	0.078*	
C5	0.78345 (15)	0.0601 (2)	0.0881 (4)	0.0654 (8)	
H5A	0.8317	0.0275	0.1237	0.078*	
C6	0.77816 (14)	0.1716 (2)	0.0729 (4)	0.0577 (7)	
H6A	0.8228	0.2141	0.1022	0.069*	
C7	0.81680 (13)	0.56925 (19)	0.0379 (3)	0.0472 (6)	
C8	0.80588 (14)	0.6767 (2)	−0.0132 (4)	0.0552 (7)	
H8A	0.7569	0.6993	−0.0642	0.066*	
C9	0.86733 (16)	0.7513 (2)	0.0109 (4)	0.0608 (7)	
H9A	0.8588	0.8240	−0.0227	0.073*	
C10	0.94103 (16)	0.7200 (3)	0.0839 (4)	0.0622 (8)	
C11	0.95105 (16)	0.6126 (3)	0.1297 (4)	0.0682 (8)	
H11A	1.0006	0.5896	0.1771	0.082*	
C12	0.89054 (14)	0.5364 (2)	0.1086 (4)	0.0608 (7)	
H12A	0.8994	0.4637	0.1416	0.073*	
C13	0.56158 (13)	0.19811 (19)	−0.1101 (3)	0.0420 (5)	
C14	0.47468 (13)	0.3509 (2)	−0.1968 (3)	0.0495 (6)	
H14A	0.4671	0.3186	−0.3157	0.059*	
H14B	0.4829	0.4289	−0.2126	0.059*	
C15	0.40087 (15)	0.3347 (2)	−0.0995 (4)	0.0617 (8)	
H15A	0.3555	0.3523	−0.1806	0.074*	
H15B	0.3968	0.2580	−0.0676	0.074*	
C16	1.0073 (2)	0.8027 (3)	0.1157 (5)	0.0921 (11)	
H16A	1.0569	0.7649	0.1374	0.138*	
H16B	0.9968	0.8470	0.2184	0.138*	
H16C	1.0101	0.8486	0.0113	0.138*	

### Atomic displacement parameters (Å^2^)

**Table:**

	*U*^11^	*U*^22^	*U*^33^	*U*^12^	*U*^13^	*U*^23^
N1	0.0424 (10)	0.0407 (11)	0.0617 (13)	−0.0019 (9)	0.0007 (9)	−0.0026 (9)
N2	0.0432 (11)	0.0418 (11)	0.0585 (13)	−0.0002 (9)	−0.0026 (9)	−0.0039 (9)
N4	0.0368 (10)	0.0412 (11)	0.0581 (13)	0.0021 (8)	−0.0039 (9)	−0.0023 (9)
O1	0.0554 (10)	0.0464 (10)	0.0678 (12)	−0.0062 (8)	−0.0189 (9)	−0.0037 (8)
O2	0.0477 (10)	0.0882 (14)	0.0579 (12)	0.0226 (9)	−0.0101 (8)	−0.0129 (10)
N3	0.0373 (10)	0.0382 (11)	0.0796 (15)	0.0020 (8)	−0.0072 (10)	−0.0020 (10)
C1	0.0409 (12)	0.0430 (13)	0.0461 (13)	0.0018 (10)	0.0015 (10)	0.0002 (10)
C2	0.0438 (12)	0.0405 (13)	0.0428 (13)	0.0027 (10)	0.0001 (10)	−0.0001 (10)
C3	0.0560 (15)	0.0425 (14)	0.0654 (17)	−0.0017 (11)	−0.0071 (12)	−0.0009 (12)
C4	0.0697 (18)	0.0407 (14)	0.082 (2)	0.0094 (13)	−0.0100 (15)	0.0012 (14)
C5	0.0509 (15)	0.0536 (16)	0.090 (2)	0.0143 (12)	−0.0097 (14)	0.0021 (15)
C6	0.0426 (13)	0.0525 (16)	0.0771 (19)	0.0020 (11)	−0.0053 (12)	0.0019 (13)
C7	0.0385 (12)	0.0428 (13)	0.0603 (15)	−0.0017 (10)	0.0020 (11)	−0.0070 (11)
C8	0.0420 (13)	0.0508 (15)	0.0726 (18)	0.0000 (11)	0.0029 (12)	0.0010 (13)
C9	0.0587 (16)	0.0510 (16)	0.0737 (18)	−0.0109 (12)	0.0113 (14)	−0.0017 (13)
C10	0.0525 (15)	0.0679 (18)	0.0669 (18)	−0.0196 (13)	0.0083 (13)	−0.0146 (15)
C11	0.0412 (14)	0.078 (2)	0.084 (2)	−0.0023 (13)	−0.0095 (13)	−0.0153 (17)
C12	0.0469 (14)	0.0481 (14)	0.086 (2)	0.0037 (12)	−0.0122 (13)	−0.0078 (14)
C13	0.0422 (12)	0.0436 (13)	0.0398 (12)	−0.0002 (10)	−0.0004 (10)	0.0004 (10)
C14	0.0487 (14)	0.0481 (14)	0.0508 (15)	0.0031 (11)	−0.0051 (11)	0.0011 (11)
C15	0.0456 (14)	0.0737 (19)	0.0643 (18)	0.0056 (13)	−0.0097 (12)	0.0006 (15)
C16	0.073 (2)	0.103 (3)	0.100 (3)	−0.043 (2)	0.0043 (18)	−0.016 (2)

### Geometric parameters (Å, °)

**Table:**

N1—N2	1.264 (3)	C6—H6A	0.9300
N1—C1	1.417 (3)	C7—C8	1.375 (4)
N2—N3	1.319 (3)	C7—C12	1.381 (3)
N4—C13	1.329 (3)	C8—C9	1.383 (3)
N4—C14	1.447 (3)	C8—H8A	0.9300
N4—H4A	0.8600	C9—C10	1.381 (4)
O1—C13	1.238 (3)	C9—H9A	0.9300
O2—C15	1.420 (3)	C10—C11	1.363 (4)
O2—H2B	0.8200	C10—C16	1.513 (4)
N3—C7	1.395 (3)	C11—C12	1.382 (4)
N3—H3A	0.8600	C11—H11A	0.9300
C1—C6	1.393 (3)	C12—H12A	0.9300
C1—C2	1.404 (3)	C14—C15	1.486 (4)
C2—C3	1.400 (3)	C14—H14A	0.9700
C2—C13	1.503 (3)	C14—H14B	0.9700
C3—C4	1.368 (3)	C15—H15A	0.9700
C3—H3B	0.9300	C15—H15B	0.9700
C4—C5	1.376 (4)	C16—H16A	0.9600
C4—H4B	0.9300	C16—H16B	0.9600
C5—C6	1.367 (4)	C16—H16C	0.9600
C5—H5A	0.9300		
			
N2—N1—C1	114.01 (19)	C10—C9—C8	121.2 (3)
N1—N2—N3	111.82 (19)	C10—C9—H9A	119.4
C13—N4—C14	122.64 (19)	C8—C9—H9A	119.4
C13—N4—H4A	118.7	C11—C10—C9	117.4 (2)
C14—N4—H4A	118.7	C11—C10—C16	121.5 (3)
C15—O2—H2B	109.5	C9—C10—C16	121.0 (3)
N2—N3—C7	121.21 (19)	C10—C11—C12	122.6 (3)
N2—N3—H3A	119.4	C10—C11—H11A	118.7
C7—N3—H3A	119.4	C12—C11—H11A	118.7
C6—C1—C2	119.0 (2)	C7—C12—C11	119.3 (3)
C6—C1—N1	123.2 (2)	C7—C12—H12A	120.3
C2—C1—N1	117.79 (19)	C11—C12—H12A	120.3
C3—C2—C1	118.0 (2)	O1—C13—N4	121.8 (2)
C3—C2—C13	115.7 (2)	O1—C13—C2	118.9 (2)
C1—C2—C13	126.3 (2)	N4—C13—C2	119.3 (2)
C4—C3—C2	121.7 (2)	N4—C14—C15	114.9 (2)
C4—C3—H3B	119.1	N4—C14—H14A	108.5
C2—C3—H3B	119.1	C15—C14—H14A	108.5
C3—C4—C5	119.9 (3)	N4—C14—H14B	108.5
C3—C4—H4B	120.1	C15—C14—H14B	108.5
C5—C4—H4B	120.1	H14A—C14—H14B	107.5
C6—C5—C4	119.8 (2)	O2—C15—C14	114.5 (2)
C6—C5—H5A	120.1	O2—C15—H15A	108.6
C4—C5—H5A	120.1	C14—C15—H15A	108.6
C5—C6—C1	121.5 (2)	O2—C15—H15B	108.6
C5—C6—H6A	119.3	C14—C15—H15B	108.6
C1—C6—H6A	119.3	H15A—C15—H15B	107.6
C8—C7—C12	119.1 (2)	C10—C16—H16A	109.5
C8—C7—N3	118.6 (2)	C10—C16—H16B	109.5
C12—C7—N3	122.3 (2)	H16A—C16—H16B	109.5
C7—C8—C9	120.3 (2)	C10—C16—H16C	109.5
C7—C8—H8A	119.8	H16A—C16—H16C	109.5
C9—C8—H8A	119.8	H16B—C16—H16C	109.5
			
C1—N1—N2—N3	178.6 (2)	N3—C7—C8—C9	179.6 (2)
N1—N2—N3—C7	−177.7 (2)	C7—C8—C9—C10	0.9 (4)
N2—N1—C1—C6	3.2 (3)	C8—C9—C10—C11	0.6 (4)
N2—N1—C1—C2	−176.6 (2)	C8—C9—C10—C16	−178.0 (3)
C6—C1—C2—C3	2.7 (3)	C9—C10—C11—C12	−1.2 (4)
N1—C1—C2—C3	−177.6 (2)	C16—C10—C11—C12	177.4 (3)
C6—C1—C2—C13	−174.8 (2)	C8—C7—C12—C11	1.3 (4)
N1—C1—C2—C13	5.0 (3)	N3—C7—C12—C11	179.8 (2)
C1—C2—C3—C4	−2.1 (4)	C10—C11—C12—C7	0.2 (5)
C13—C2—C3—C4	175.6 (2)	C14—N4—C13—O1	−6.1 (4)
C2—C3—C4—C5	−0.5 (4)	C14—N4—C13—C2	173.9 (2)
C3—C4—C5—C6	2.5 (5)	C3—C2—C13—O1	−11.0 (3)
C4—C5—C6—C1	−2.0 (5)	C1—C2—C13—O1	166.5 (2)
C2—C1—C6—C5	−0.7 (4)	C3—C2—C13—N4	169.0 (2)
N1—C1—C6—C5	179.5 (3)	C1—C2—C13—N4	−13.5 (3)
N2—N3—C7—C8	169.7 (2)	C13—N4—C14—C15	76.5 (3)
N2—N3—C7—C12	−8.8 (4)	N4—C14—C15—O2	71.8 (3)
C12—C7—C8—C9	−1.9 (4)		

### Hydrogen-bond geometry (Å, °)

**Table:**

*D*—H···*A*	*D*—H	H···*A*	*D*···*A*	*D*—H···*A*
N4—H4A···N1	0.86	2.05	2.696 (3)	132
O2—H2B···O1^i^	0.82	1.92	2.729 (2)	169
N3—H3A···O2^ii^	0.86	2.00	2.851 (2)	170

Symmetry codes: (i) *x*, −*y*+1/2, *z*+1/2; (ii) −*x*+1, −*y*+1, −*z*.
